# Supplementary material for: Histone serotonylation in dorsal raphe nucleus contributes to stress- and antidepressant-mediated gene expression and behavior
Source: Nat Commun. 2024 Jun 13;15:5042. doi: 10.1038/s41467-024-49336-4 (PMC11176395; doi:10.1038/s41467-024-49336-4)
Supplement: Supplementary file 1 — Supplementary Information [file 41467_2024_49336_MOESM1_ESM.pdf]

SUPPLEMENTARY INFORMATION

Histone serotonylation in dorsal raphe nucleus contributes to stress- and antidepressant-mediated gene expression and behavior

Amni Al-Kachak, Giuseppina Di Salvo, Sasha L. Fulton, Jennifer C Chan, Lorna A. Farrelly, Ashley E. Lepack, Ryan M. Bastle, Lingchun Kong, Flurin Cathomas, Emily L. Newman, Caroline Menard, Aarthi Ramakrishnan, Polina Safovich, Yang Lyu, Herbert E. Covington III, Li Shen, Kelly Gleason, Carol A. Tamminga, Scott J. Russo, Ian Maze

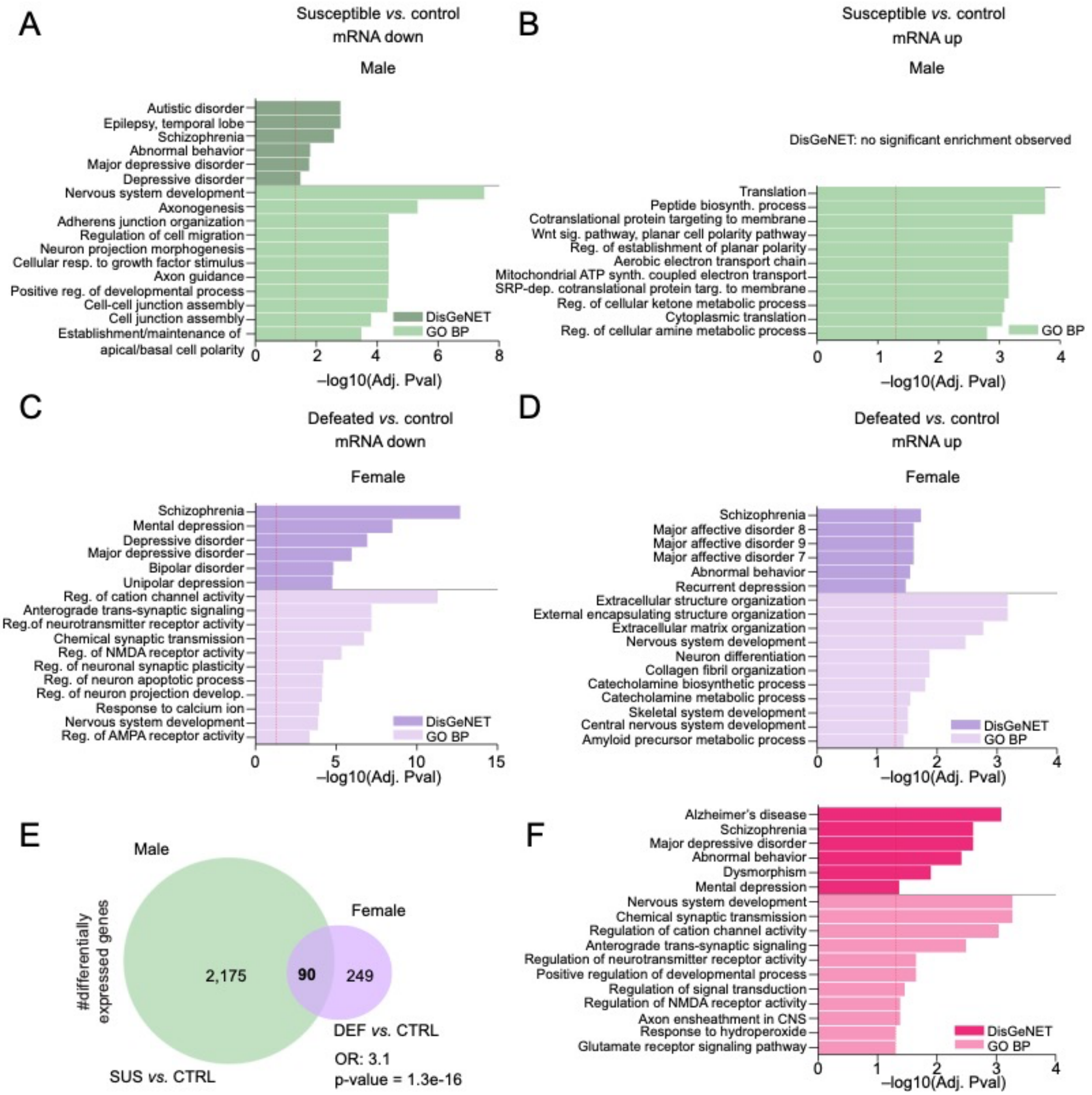

Supplementary Figure 1. Directionality and overlap of gene expression changes observed following CSDS in males *vs.* females.

(A) Example GO Biological Process and DisGeNET pathway enrichment (FDR<0.05; Benjamini-Hochberg) for the PCGs significantly downregulated (at FDR<0.1) in susceptible *vs.* control males. Dashed line indicates significance via adjusted p-value. **(B)** Example GO Biological Process and DisGeNET pathway enrichment (FDR<0.05; Benjamini-Hochberg) for the PCGs

significantly upregulated (at FDR<0.1) in susceptible *vs.* control males. Dashed line indicates significance via adjusted p-value. **(C)** Example GO Biological Process and DisGeNET pathway enrichment (FDR<0.05; Benjamini-Hochberg) for the PCGs significantly downregulated (at FDR<0.1) in defeated *vs.* control females. Dashed line indicates significance via adjusted p-value. **(D)** Example GO Biological Process and DisGeNET pathway enrichment (FDR<0.05; Benjamini-Hochberg) for the PCGs significantly upregulated (at FDR<0.1) in defeated *vs.* control females. Dashed line indicates significance via adjusted p-value. **(E)** Venn diagram depicting the overlap (independent of directionality) between differentially expressed PCGs in male susceptible *vs.* control and female defeated *vs.* control. Odds ratio (OR) and respective p-value (Fisher's exact test) of overlap is provided. **(F)** Example GO Biological Process and DisGeNET pathway enrichment (FDR<0.05; Benjamini-Hochberg) for the differentially expressed PCGs significantly overlapping between male *vs.* female.

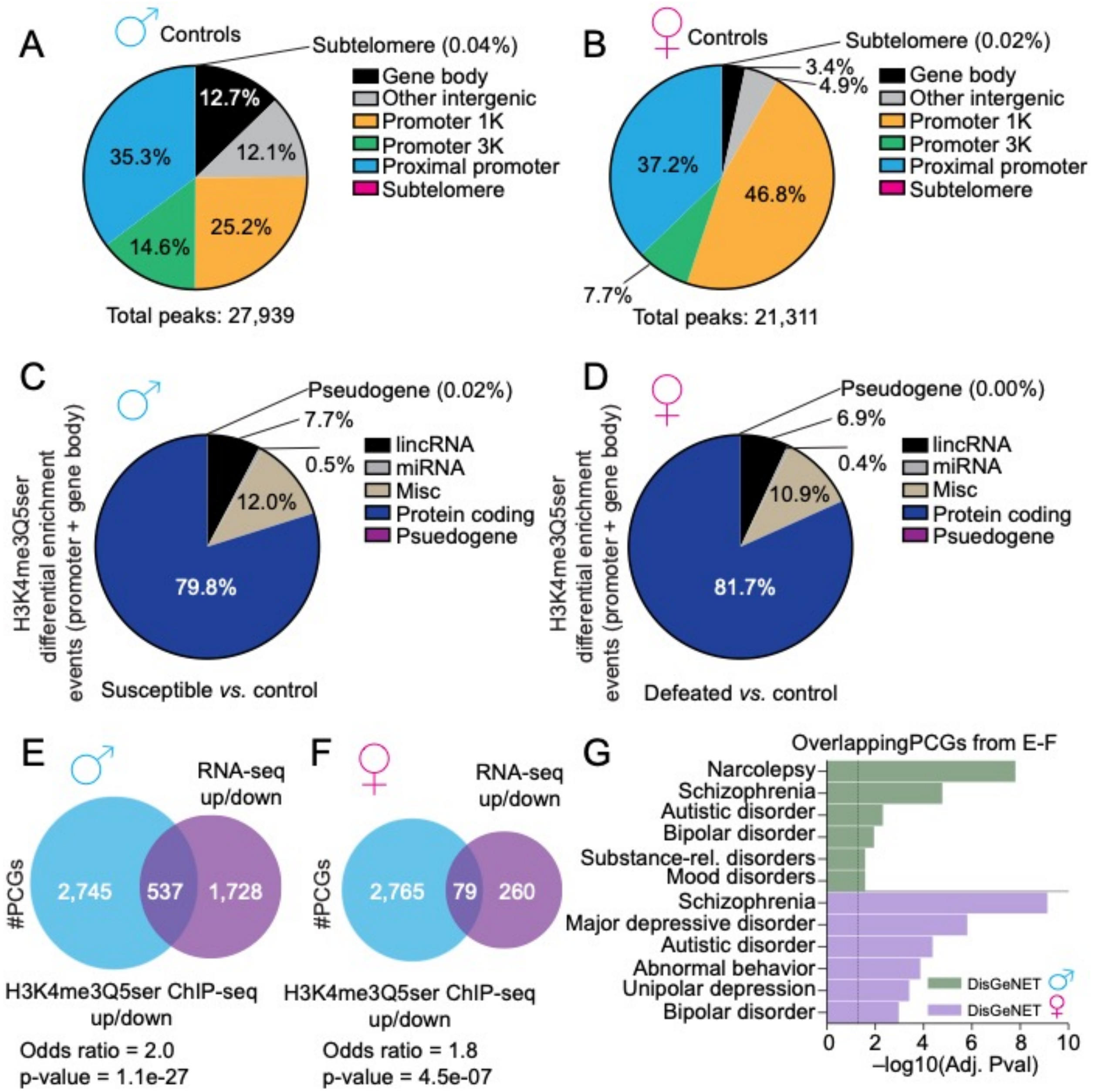

Supplementary Figure 2. Genomic distributions of H3K4me3Q5ser baseline and differential enrichment, and correlations with differential gene expression.

Pie charts describing the genomic distribution of H3K4me3Q5ser peaks (FDR<0.05, >5-fold enrichment over DNA input) in control **(A)** male *vs.* **(B)** female DRN. Pie charts describing the genomic distribution of H3K4me3Q5ser differential enrichment events (FDR<0.05, $\geq$1.5 or $\leq$ –1.5) in promoters and gene bodies of **(C)** male susceptible *vs.* control or **(D)** female defeated *vs.*

control DRN broken down by gene ‘type.’ Venn diagrams describing the overlap between differentially expressed PCGs (FDR<0.1, irrespective of directionality) and PCGs displaying differential H3K4me3Q5ser enrichment (FDR<0.05, $\geq$ 1.5 or $\leq$ –1.5 fold change, irrespective of directionality) in **(E)** male susceptible *vs.* control or **(F)** female defeated *vs.* control DRN. Odds ratios (OR) and respective p-values (Fisher’s exact test) of overlap are provided. **(G)** Example DisGeNET pathway enrichment (FDR<0.05; Benjamini-Hochberg) for overlapping PCGs identified in E-F for males and females. Dashed line indicates significance via adjusted p-value.

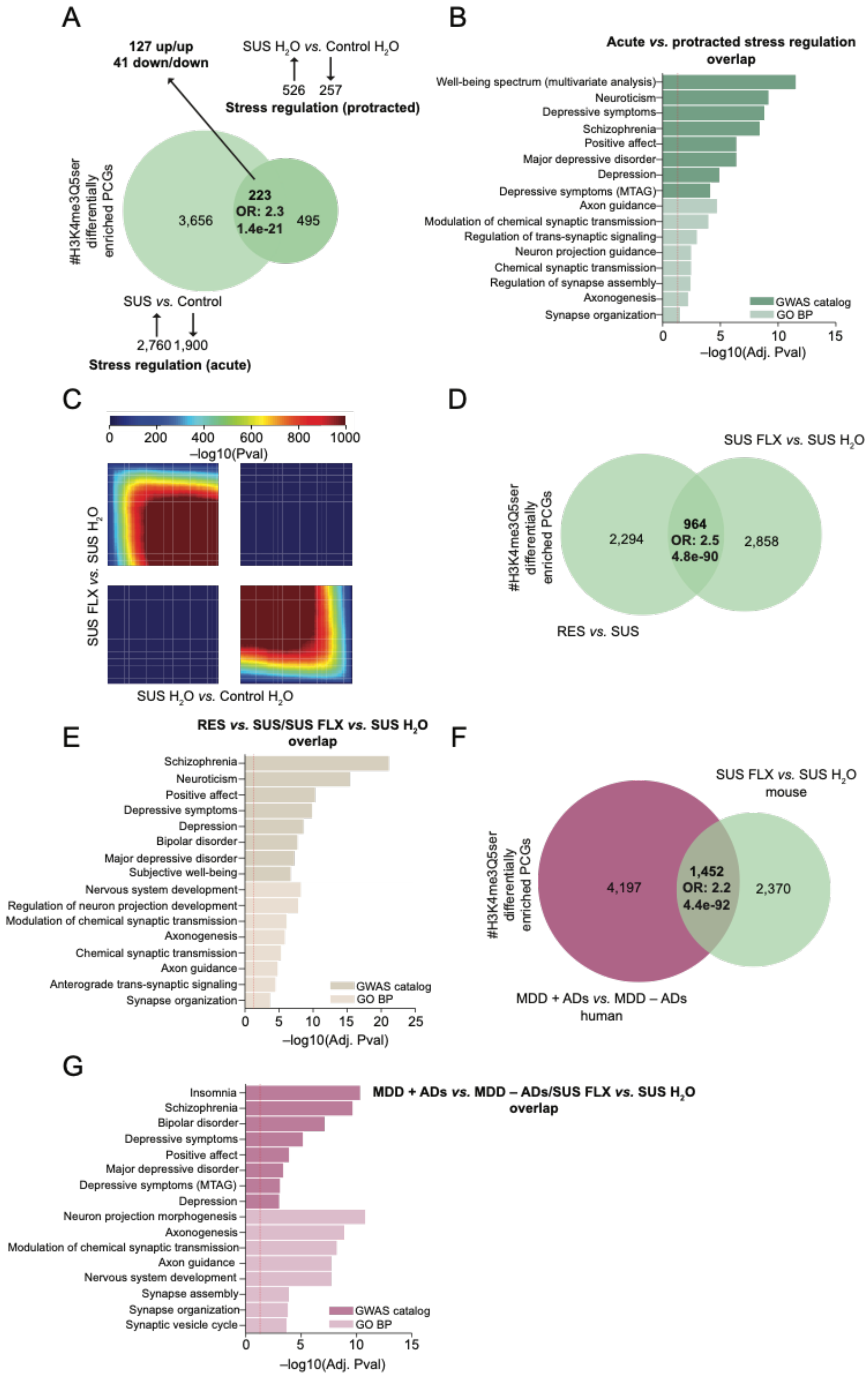
A
127 up/up
41 down/down
SUS H2O vs. Control H2O
526 257
Stress regulation (protracted)
#H3K4me3Q5ser differentially enriched PCGs
3,656
223
OR: 2.3
1.4e-21
495
SUS vs. Control
2,760 1,900
Stress regulation (acute)
B
Acute vs. protracted stress regulation overlap
Well-being spectrum (multivariate analysis)
Neuroticism
Depressive symptoms
Schizophrenia
Positive affect
Major depressive disorder
Depression
Depressive symptoms (MTAG)
Axon guidance
Modulation of chemical synaptic transmission
Regulation of trans-synaptic signaling
Neuron projection guidance
Chemical synaptic transmission
Regulation of synapse assembly
Axonogenesis
Synapse organization
GWAS catalog
GO BP
–log10(Adj. Pval)
C
–log10(Pval)
SUS FLX vs. SUS H2O
SUS H2O vs. Control H2O
D
SUS FLX vs. SUS H2O
#H3K4me3Q5ser differentially enriched PCGs
2,294
964
OR: 2.5
4.8e-90
2,858
RES vs. SUS
E
RES vs. SUS/SUS FLX vs. SUS H2O overlap
Schizophrenia
Neuroticism
Positive affect
Depressive symptoms
Depression
Bipolar disorder
Major depressive disorder
Subjective well-being
Nervous system development
Regulation of neuron projection development
Modulation of chemical synaptic transmission
Axonogenesis
Chemical synaptic transmission
Axon guidance
Anterograde trans-synaptic signaling
Synapse organization
GWAS catalog
GO BP
–log10(Adj. Pval)
F
SUS FLX vs. SUS H2O mouse
#H3K4me3Q5ser differentially enriched PCGs
4,197
1,452
OR: 2.2
4.4e-92
2,370
MDD + ADs vs. MDD – ADs human
G
MDD + ADs vs. MDD – ADs/SUS FLX vs. SUS H2O overlap
Insomnia
Schizophrenia
Bipolar disorder
Depressive symptoms
Positive affect
Major depressive disorder
Depressive symptoms (MTAG)
Depression
Neuron projection morphogenesis
Axonogenesis
Modulation of chemical synaptic transmission
Axon guidance
Nervous system development
Synapse assembly
Synapse organization
Synaptic vesicle cycle
GWAS catalog
GO BP
–log10(Adj. Pval)

Supplementary Figure 3. Chronic fluoxetine in CSDS male mice regulates H3K4me3Q5ser dynamics in DRN at PCGs relevant to stress-resilience and AD responsiveness in humans with MDD.

(A) Venn diagram depicting the overlap (independent of directionality) between PCGs differentially enriched for H3K4me3Q5ser in male susceptible *vs.* control (24 hr post-SI testing; acute stress regulation) and male susceptible H_2O *vs.* control H_2O mice (30d post-initial SI testing; protracted stress regulation). Odds ratio (OR) and respective p-value (Fisher's exact test) of overlap is provided. **(B)** Example GO Biological Process and GWAS Catalog pathway enrichment (FDR<0.05; Benjamini-Hochberg) for the differentially enriched PCGs significantly overlapping between the two comparisons in A. **(C)** Threshold-free RRHO analyses comparing transcriptional profiles for stress-regulated genes in the absence or presence of fluoxetine at protracted periods following CSDS (n = 4-5/group), demonstrating that chronic fluoxetine treatments significantly reversed gene expression programs observed in response to stress in vehicle treated animals. Each pixel represents the overlap between differential transcriptomes, with the significance of overlap of a hypergeometric test color-coded. **(D)** Venn diagram depicting the overlap (independent of directionality) between PCGs differentially enriched for H3K4me3Q5ser in male resilient *vs.* stress-susceptible (24 hr post-SI testing) and male susceptible FLX *vs.* susceptible H_2O mice (30d post-initial SI testing). Odds ratio (OR) and respective p-value (Fisher's exact test) of overlap is provided. **(E)** Example GO Biological Process and GWAS Catalog pathway enrichment (FDR<0.05; Benjamini-Hochberg) for the differentially enriched PCGs significantly overlapping between the two comparisons in D. **(F)** Venn diagram depicting the overlap (independent of directionality) between PCGs differentially enriched for H3K4me3Q5ser in male susceptible FLX *vs.* susceptible H_2O (30d hr post-initial SI testing) and human MDD subjects -/+ ADs onboard at their time of death (n = 5/group). Odds ratio (OR) and respective p-value (Fisher's exact test) of

overlap is provided. **(G)** Example GO Biological Process and GWAS Catalog pathway enrichment (FDR<0.05; Benjamini-Hochberg) for the differentially enriched PCGs significantly overlapping between the two comparisons in F.

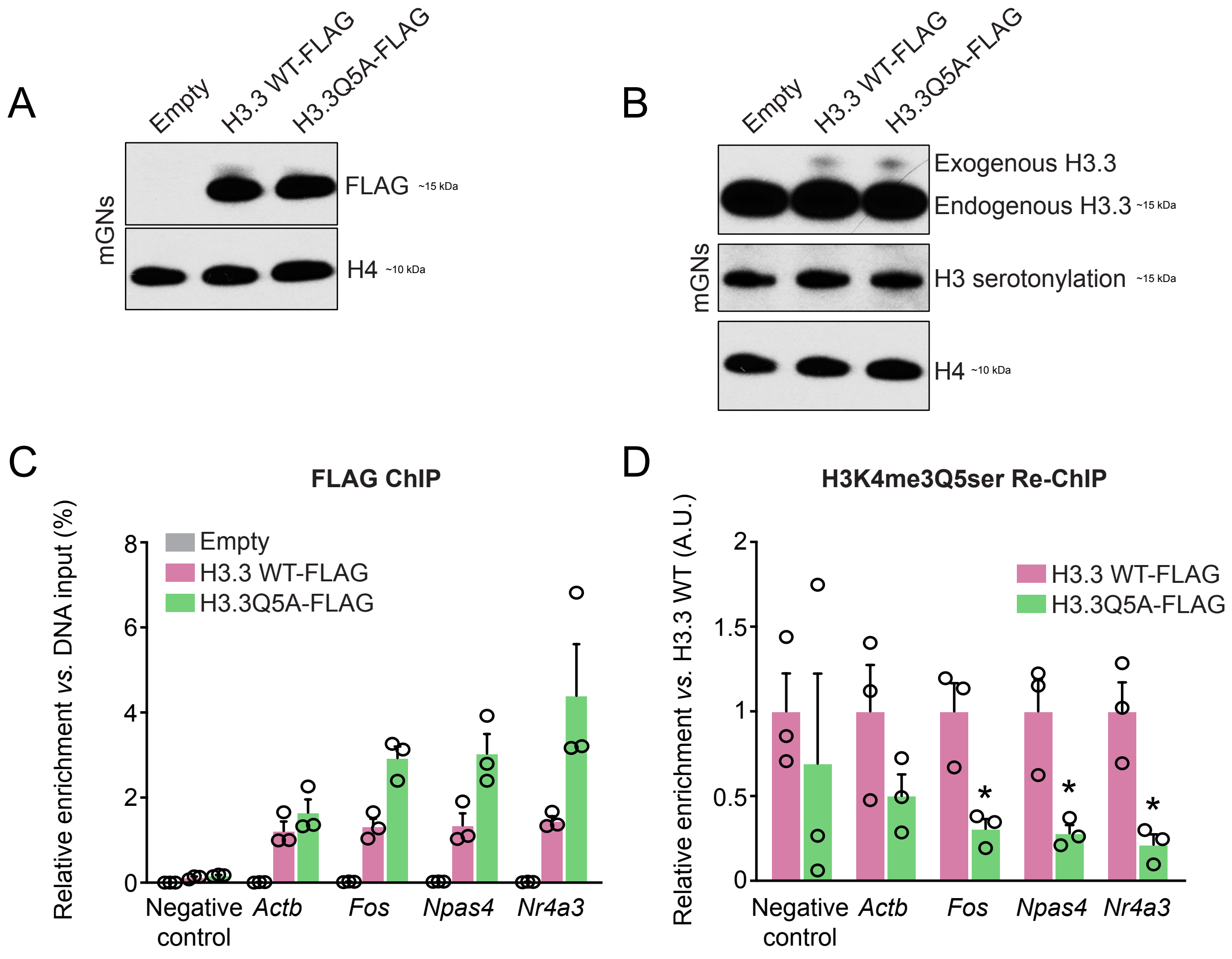

Supplementary Figure 4. Validation of H3K4me3Q5ser downregulation in primary neurons at H3.3Q5A incorporated loci.

(A) Western blotting validation of H3.3 WT-FLAG *vs.* H3.3Q5A-FLAG (*vs.* empty vector) expression – assessed using an anti-FLAG antibody – in cultured cerebellar granule neurons (cGNs). Total H4 was used as a loading control. **(B)** Western blotting-based comparison of endogenous *vs.* exogenous H3.3 expression (this antibody recognizes the globular domain of H3.3 and therefore can recognize both endogenous and exogenous H3.3) following transduction with empty *vs.* H3.3 WT-FLAG *vs.* H3.3Q5A-FLAG vectors in cGNs. Note that transduction with the aforementioned viruses (which account for ~5% of the total H3.3 pool) did not result in a global downregulation of H3 serotonylation in these cells. Total H4 was used as a loading control. **(C)**

FLAG ChIP-qPCRs following transduction with empty *vs.* H3.3 WT-FLAG *vs.* H3.3Q5A-FLAG vectors in cGNs demonstrating incorporation of exogenously expressed H3.3 (no signal observed in empty vector control expressing cells) proteins within the promoter of permissive genes but not within a negative control genomic locus. **(D)** FLAG ChIP-/H3K4me3Q5ser re-ChIP-qPCRs following transduction with empty *vs.* H3.3 WT-FLAG *vs.* H3.3Q5A-FLAG vectors in cGNs demonstrating significant loss of the serotonylation mark at H3.3Q5A *vs.* H3.3 WT incorporated genes, but not within a negative control locus (Student's two-tailed t tests – Actb: p=0.1742, t_4 = 1.650; *Fos*: p=0.0180, t_4 = 3.939; *Npas4*: p=0.0210, t_4 = 3.689; *Nr4a3*: p=0.0123, t_4 = 4.335) empty vector ChIP-/re-ChIPs were excluded from this analysis based upon the lack of enrichment observed for this vector in C. Data presented as mean +/- SEM. A.U., arbitrary units; in D, data were normalized to respective enrichment of H3.3Q5A-FLAG *vs.* H3.3 WT-FLAG vectors to control for incorporation rates. For all bar graphs, data presented as mean ± SEM. Source data are provided as a Source Data file.

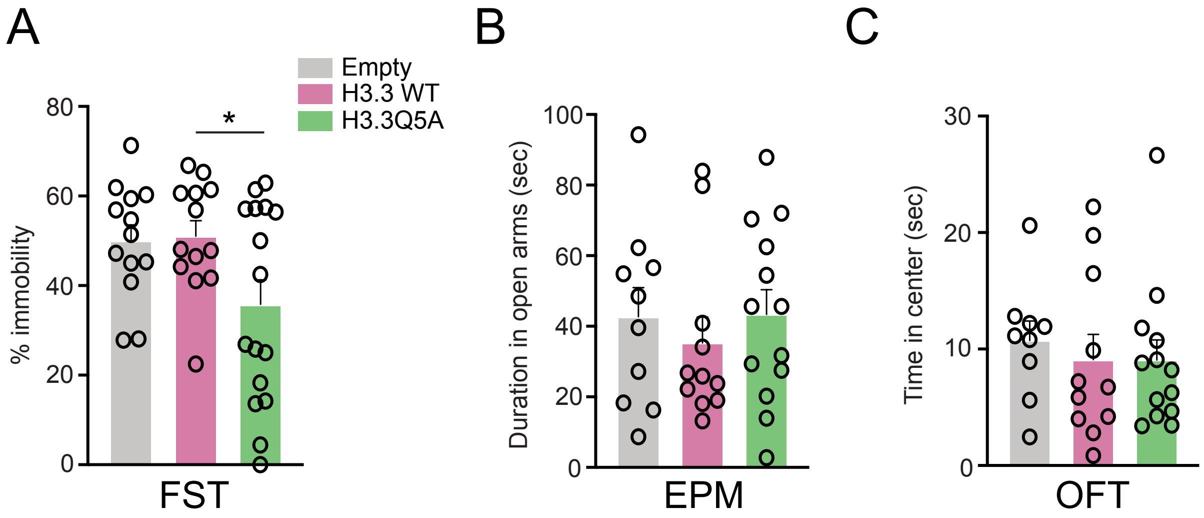

Supplementary Figure 5. Reducing H3 serotonylation in DRN of an independent cohort of non-stressed mice reduces behavioral despair, but does not affect anxiety-related behaviors.

(A) Percent immobility in the forced swim test (FST) for control (i.e., non-stressed) male mice virally transduced intra-DRN with empty *vs.* H3.3WT *vs.* H3.3Q5A vectors (n = 13-16/group). Data were analyzed using a one-way ANOVA, with significant main effects (p=0.0319, $F_{2,39}$ = 3.768). Tukey's multiple comparisons test revealed significant differences between H3.3 WT *vs.* H3.3Q5A-transduced mice (p=0.05), with trending effects observed comparing empty *vs.* H3.3Q5A-transduced mice (p=0.07). **(B)** Duration of time spent in the open arms in the elevated plus maze (EPM) for control (i.e., non-stressed) male mice virally transduced intra-DRN with empty *vs.* H3.3WT *vs.* H3.3Q5A vectors (n = 10-13/group). Data were analyzed using a one-way ANOVA, with no significant main effects observed (p>0.05, $F_{2,31}$ = 0.3605). **(C)** Duration of time spent in the center in the open field test (OFT) for control (i.e., non-stressed) male mice virally transduced intra-DRN with empty *vs.* H3.3WT *vs.* H3.3Q5A vectors (n = 9-13/group). Data were analyzed using a one-way ANOVA, with no significant main effects observed (p>0.05, $F_{2,30}$ = 0.2260). Data presented as mean (+/- SEM). For all bar graphs, data presented as mean ± SEM. Source data are provided as a Source Data file.

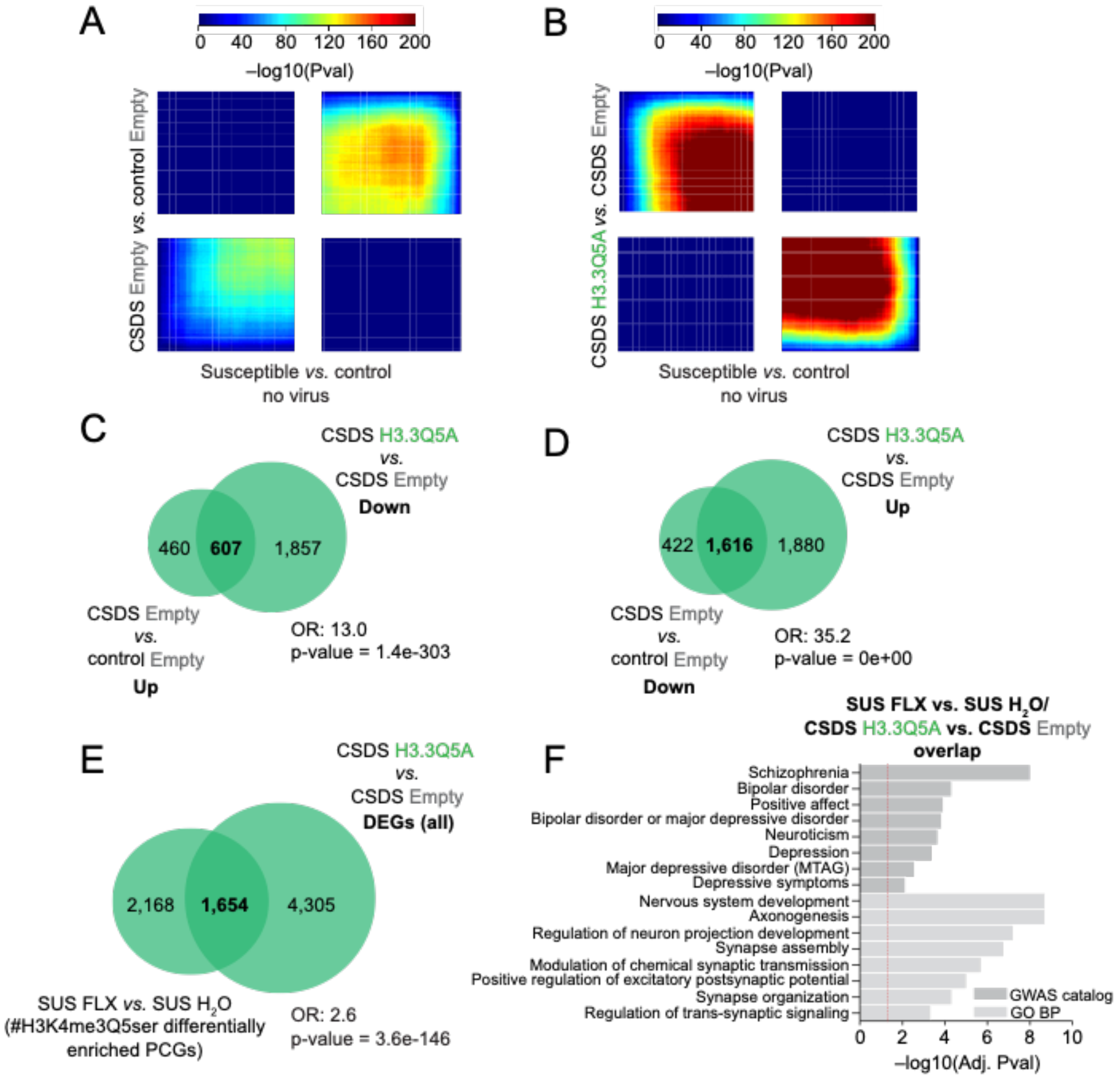

Supplementary Figure 6. Correlations between gene expression profiles in DRN from susceptible *vs.* control male mice, -/+ viral transduction, and reversal of stress-induced gene expression in H3.3Q5A transduced animals.

(A) Threshold-free RRHO analyses comparing transcriptional profiles for stress-regulated genes in empty vector (CSDS *vs.* control; *n* = 7-8/group) to non-virally transduced DRN from CSDS mice (*n* = 7-8/group). **(B)** Threshold-free RRHO analyses comparing transcriptional profiles for stress-regulated genes in susceptible *vs.* control (no virus; *n* = 7-8/group) male mice to H3.3Q5A

CSDS *vs.* empty CSDS (*n* = 8-9/group). For A-B, each pixel represents the overlap between differential transcriptomes, with the significance of overlap of a hypergeometric test color-coded. Venn diagrams of overlap between PCGs displaying significant (**C**) upregulation (FDR<0.1) or **(D)** downregulation in their expression in CSDS empty *vs.* control empty comparisons and reversal of stress-induced gene expression following transduction with H3.3Q5A. Odds ratios (OR) and respective p-values (Fisher's exact test) of overlap are provided. **(E)** Venn diagram depicting the overlap (independent of directionality) between PCGs differentially enriched for H3K4me3Q5ser in male susceptible FLX *vs.* susceptible H_2O mice (30d post-initial SI testing) and differentially expressed genes between H3.3Q5A CSDS *vs.* empty CSDS animals. Odds ratio (OR) and respective p-value (Fisher's exact test) of overlap is provided. **(F)** Example GO Biological Process and GWAS Catalog pathway enrichment (FDR<0.05; Benjamini-Hochberg) for the differentially enriched PCGs significantly overlapping between the two comparisons in E.

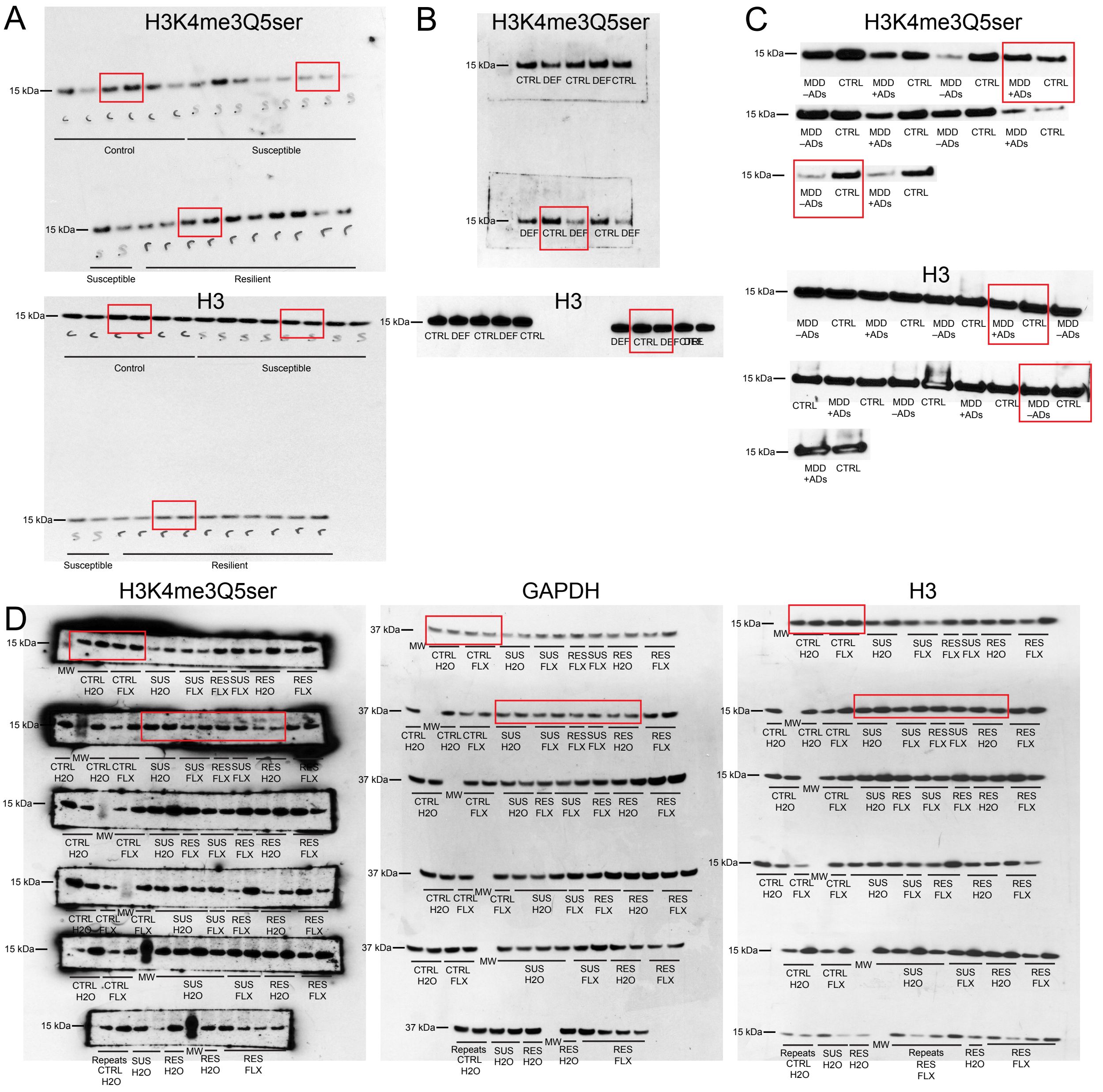

Supplementary Figure 7. Uncropped western blots from Figures 2 and 3.

(A) Uncropped western blots used for quantifications in Figure 2A. **(B)** Uncropped western blots used for quantifications in Figure 2B. **(C)** Uncropped western blots used for quantifications in Figure 2C. **(D)** Uncropped western blots used for quantifications in Figure 3D. Red rectangles indicate the representative blots displayed in the main Figures.

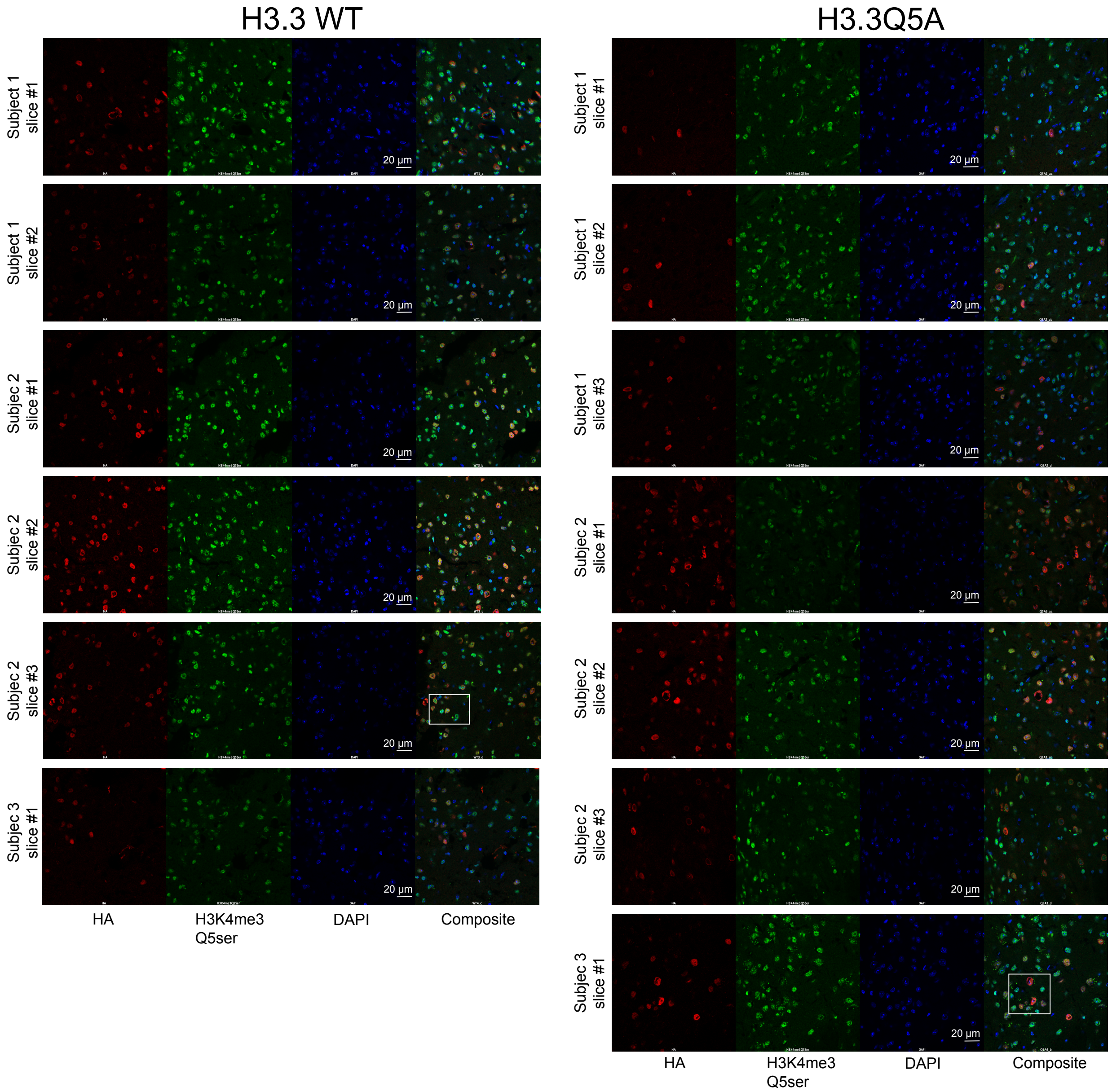

Supplementary Figure 8. Immunofluorescence images quantified in Fig. 4B.

White rectangles indicate the representative images displayed in the main Figure.
