# Supplementary material for: Histone serotonylation in dorsal raphe nucleus contributes to stress- and antidepressant-mediated gene expression and behavior
Source: Nat Commun. 2024 Jun 13;15:5042. doi: 10.1038/s41467-024-49336-4 (PMC11176395; doi:10.1038/s41467-024-49336-4)
Supplement: Supplementary file 3 — Description of Additional Supplementary Files [file 41467_2024_49336_MOESM3_ESM.pdf]

DESCRIPTION OF ADDITIONAL SUPPLEMENTARY FILES

Excel file including Supplementary Data 1-71, which contain processed ChIP-seq and RNA-seq from adult male and female DRN, as well as human demographic information and mouse ChIP-qPCR primers.

Supplementary Data 1: Adult DRN (male) RNA-seq data (Susceptible vs. Control All), FDR<0.1 (DESeq2) – Fig. 1B-C & Fig. S1A-B, E-F

Supplementary Data 2: Adult DRN (male) RNA-seq data (Susceptible vs. Control All), FDR<0.1, DisGeNET (FDR<0.05; Benjamini-Hochberg) – Fig. 1C

Supplementary Data 3: Adult DRN (male) RNA-seq data (Susceptible vs. Control All), FDR<0.1, Go BP (FDR<0.05; Benjamini-Hochberg) – Fig. 1C

Supplementary Data 4: Adult DRN (male) RNA-seq data (Susceptible vs. Control Down), FDR<0.1, DisGeNET (FDR<0.05; Benjamini-Hochberg) –Fig. S1A

Supplementary Data 5: Adult DRN (male) RNA-seq data (Susceptible vs. Control Up), FDR<0.1, DisGeNET (FDR<0.05; Benjamini-Hochberg) –Fig. S1B

Supplementary Data 6: Adult DRN (male) RNA-seq data (Susceptible vs. Control Down), FDR<0.1, GO BP (FDR<0.05; Benjamini-Hochberg) –Fig. S1A

Supplementary Data 7: Adult DRN (male) RNA-seq data (Susceptible vs. Control Up), FDR<0.1, GO BP (FDR<0.05; Benjamini-Hochberg) –Fig. S1B

Supplementary Data 8: Adult DRN (male) RNA-seq data (Susceptible vs. Resilient All), FDR<0.1 (DESeq2) – Fig. 1B

Supplementary Data 9: Adult DRN (male) RNA-seq data (Resilient vs. Control All), FDR<0.1 (DESeq2) – Fig. 1B

Supplementary Data 10: Adult DRN (female) RNA-seq data (Defeated vs. Control All), FDR<0.1 (DESeq2) – Fig. 1E-F & Fig. S1C-F

Supplementary Data 11: Adult DRN (female) RNA-seq data (Defeated vs. Control All), FDR<0.1, DisGeNET (FDR<0.05; Benjamini-Hochberg) – Fig. 1F

Supplementary Data 12: Adult DRN (female) RNA-seq data (Defeated vs. Control All), FDR<0.1, Go BP (FDR<0.05; Benjamini-Hochberg) – Fig. 1F

Supplementary Data 13: Adult DRN (female) RNA-seq data (Defeated vs. Control Down), FDR<0.1, DisGeNET (FDR<0.05; Benjamini-Hochberg) –Fig. S1C

Supplementary Data 14: Adult DRN (female) RNA-seq data (Defeated vs. Control Up), FDR<0.1, DisGeNET (FDR<0.05; Benjamini-Hochberg) –Fig. S1D

Supplementary Data 15: Adult DRN (female) RNA-seq data (Defeated vs. Control Down), FDR<0.1, GO BP (FDR<0.05; Benjamini-Hochberg) –Fig. S1C

Supplementary Data 16: Adult DRN (female) RNA-seq data (Defeated vs. Control Up), FDR<0.1, GO BP (FDR<0.05; Benjamini-Hochberg) –Fig. S1D

Supplementary Data 17: Adult DRN RNA-seq data (Male Susceptible vs. Control & Female Defeated vs. Control Overlap All), FDR<0.1, DisGeNET (FDR<0.05; Benjamini-Hochberg) –Fig. S1F

Supplementary Data 18: Adult DRN RNA-seq data (Male Susceptible vs. Control & Female Defeated vs. Control Overlap All), FDR<0.1, Go BP (FDR<0.05; Benjamini-Hochberg) –Fig. S1F

Supplementary Data 19: Adult DRN (male) H3K4me3Q5ser ChIP-seq peaks (Control), FDR<0.05 (MACS2), >5-fold enrichment over input – Figure 2D-I & Fig. S2A-B

Supplementary Data 20: Adult DRN (male) H3K4me3Q5ser ChIP-seq peaks (Susceptible), FDR<0.05 (MACS2), >5-fold enrichment over input – Figure 2D-I & Fig. S2A-B

Supplementary Data 21: Adult DRN (male) H3K4me3Q5ser ChIP-seq peaks (Resilient), FDR<0.05 (MACS2), >5-fold enrichment over input – Figure 2D-I & Fig. S2A-B

Supplementary Data 22: Adult DRN (female) H3K4me3Q5ser ChIP-seq peaks (Control), FDR<0.05 (MACS2), >5-fold enrichment over input – Figure 2D-G & Fig. S2A-B

Supplementary Data 23: Adult DRN (female) H3K4me3Q5ser ChIP-seq peaks (Defeated), FDR<0.05 (MACS2), >5-fold enrichment over input – Figure 2D-G & Fig. S2A-B

Supplementary Data 24: Adult DRN (male) H3K4me3Q5ser diffReps (Susceptible vs. Control All, FDR<0.05, log2FC ≥ 1.5 or ≤ –1.5 – Fig. 2F-G & Fig. S2C, E, G

Supplementary Data 25: Adult DRN (male) H3K4me3Q5ser diffReps (Susceptible vs. Resilient All, FDR<0.05, log2FC ≥ 1.5 or ≤ –1.5 – Fig. 2H-I & Fig. S2C, E, G

Supplementary Data 26: Adult DRN (male) H3K4me3Q5ser diffReps (Resilient vs. Control All, FDR<0.05, log2FC ≥ 1.5 or ≤ –1.5 – Fig. 2

Supplementary Data 27: Adult DRN (female) H3K4me3Q5ser diffReps (Defeated vs. Control All, FDR<0.05, log2FC ≥ 1.5 or ≤ –1.5 – Fig. 2F-G & Fig. S2D, F, G

Supplementary Data 28: Adult DRN ChIP-seq data (Male Susceptible vs. Control & Female Defeated vs. Control Overlap All), FDR<0.05, log2FC ≥ 1.5 or ≤ –1.5, GWAS Catalog (FDR<0.05; Benjamini-Hochberg) – Fig. 2G

Supplementary Data 29: Adult DRN ChIP-seq data (Male Susceptible vs. Control & Female Defeated vs. Control Overlap All), FDR<0.05, log2FC ≥ 1.5 or ≤ –1.5, DisGeNET (FDR<0.05; Benjamini-Hochberg) – Fig. 2G

Supplementary Data 30: Adult DRN ChIP-seq data (Male Susceptible vs. Control & Female Defeated vs. Control Overlap All), FDR<0.05, log2FC ≥ 1.5 or ≤ –1.5, GO BP (FDR<0.05; Benjamini-Hochberg) – Fig. 2G

Supplementary Data 31: Adult DRN ChIP-seq data (Male Susceptible vs. Control & Male Resilient vs. Susceptible Overlap, Reversed in Resilient), FDR<0.05, log2FC ≥ 1.5 or ≤ –1.5, GWAS Catalog (FDR<0.05; Benjamini-Hochberg) – Fig. 2I

Supplementary Data 32: Adult DRN ChIP-seq data (Male Susceptible vs. Control & Male Resilient vs. Susceptible Overlap, Reversed in Resilient), FDR<0.05, log2FC ≥ 1.5 or ≤ –1.5, DisGeNET (FDR<0.05; Benjamini-Hochberg) – Fig. 2I

Supplementary Data 33: Adult DRN ChIP-seq data (Male Susceptible vs. Control & Male Resilient vs. Susceptible Overlap, Reversed in Resilient), FDR<0.05, log2FC ≥ 1.5 or ≤ –1.5, GO BP (FDR<0.05; Benjamini-Hochberg) – Fig. 2I

Supplementary Data 34: Adult DRN ChIP-seq data (Male Susceptible vs. Control All; FDR<0.05, log2FC ≥ 1.5 or ≤ –1.5) vs. Adult DRN RNA-seq data (Male Susceptible vs. Control All; FDR<0.1), DisGeNET (FDR<0.05; Benjamini-Hochberg) –Fig. S2G (Overlapping PCGs)

Supplementary Data 35: Adult DRN ChIP-seq data (Female Defeated vs. Control All; FDR<0.05, log2FC ≥ 1.5 or ≤ –1.5) vs. Adult DRN RNA-seq data (Female Defeated vs. Control All; FDR<0.1), DisGeNET (FDR<0.05; Benjamini-Hochberg) –Fig. S2G (Overlapping PCGs)

Supplementary Data 36: Adult DRN (male) H3K4me3Q5ser ChIP-seq peaks (Control H2O), FDR<0.05 (MACS2), >5-fold enrichment over input – Figure 3E-G & Fig. S3A-B

Supplementary Data 37: Adult DRN (male) H3K4me3Q5ser ChIP-seq peaks (Susceptible H2O), FDR<0.05 (MACS2), >5-fold enrichment over input – Figure 3E-H & Fig. S3A-B, D-E

Supplementary Data 38: Adult DRN (male) H3K4me3Q5ser diffReps (Susceptible H2O vs. Control H2O All, FDR<0.05, log2FC ≥ 1.0 or ≤ –1.0 – Fig. 3E-G & Fig. S3A-B

Supplementary Data 39: Adult DRN (male) H3K4me3Q5ser diffReps (Susceptible vs. Control All, FDR<0.05, log2FC ≥ 1.0 or ≤ –1.0 – Fig. 3E-G & Fig. S3A-B

Supplementary Data 40: Adult DRN ChIP-seq data (Male Susceptible vs. Control Acute & Male Susceptible vs. Control Protracted Overlap All), FDR<0.05, log2FC ≥ 1.0 or ≤–1.0, GO BP (FDR<0.05; Benjamini-Hochberg) –Fig. S3B

Supplementary Data 41: Adult DRN ChIP-seq data (Male Susceptible vs. Control Acute & Male Susceptible vs. Control Protracted Overlap All), FDR<0.05, log2FC ≥ 1.0 or ≤–1.0, GWAS Catalog (FDR<0.05; Benjamini-Hochberg) –Fig. S3B

Supplementary Data 42: Adult DRN (male) RNA-seq data (Susceptible H2O vs. Control H2O All), –Fig. S3C

Supplementary Data 43: Adult DRN (male) RNA-seq data (Susceptible FLX vs. Control H2O All; DESeq2), –Fig. S3C

Supplementary Data 44: Adult DRN (male) H3K4me3Q5ser ChIP-seq peaks (Control FLX), FDR<0.05 (MACS2), >5-fold enrichment over input – Figure 3E-F

Supplementary Data 45: Adult DRN (male) H3K4me3Q5ser ChIP-seq peaks (Susceptible FLX), FDR<0.05 (MACS2), >5-fold enrichment over input – Figure 3E-H & Fig. S3D-G

Supplementary Data 46: Adult DRN (male) H3K4me3Q5ser diffReps (Susceptible FLX vs. Susceptible H2OAll, FDR<0.05, log2FC ≥ 1.0 or ≤ –1.0 – Fig. 3E-H & Fig. S3D-G

Supplementary Data 47: Adult DRN (male) H3K4me3Q5ser diffReps (Control FLX vs. Control H2O All, FDR<0.05, log2FC ≥ 1.0 or ≤ –1.0 – Fig. 3E-F

Supplementary Data 48: Adult (Male) DRN ChIP-seq data (Susceptible FLX vs. Susceptible H2O All), FDR<0.05, log2FC ≥ 1.0 or ≤–1.0, GO BP (FDR<0.05; Benjamini-Hochberg) – Fig. 3H

Supplementary Data 49: Adult (Male) DRN ChIP-seq data (Susceptible FLX vs. Susceptible H2O All), FDR<0.05, log2FC ≥ 1.0 or ≤–1.0, GWAS Catalog (FDR<0.05; Benjamini-Hochberg) – Fig. 3H

Supplementary Data 50: Adult DRN (male) H3K4me3Q5ser diffReps (Susceptible vs. Resilient All, FDR<0.05, log2FC ≥ 1.0 or ≤ –1.0 –Fig. S3D-E

Supplementary Data 51: Adult (Male) DRN ChIP-seq data (Susceptible FLX vs. Susceptible H2O & Resilient vs. Susceptible Overlap All), FDR<0.05, log2FC ≥ 1.0 or ≤–1.0, GO BP (FDR<0.05; Benjamini-Hochberg) –Fig. S3E

Supplementary Data 52: Adult (Male) DRN ChIP-seq data (Susceptible FLX vs. Susceptible H2O & Resilient vs. Susceptible Overlap All), FDR<0.05, log2FC ≥ 1.0 or ≤–1.0, GWAS Catalog (FDR<0.05; Benjamini-Hochberg) –Fig. S3E

Supplementary Data 53: Human DRN H3K4me3Q5ser ChIP-seq peaks (Matched Controls for MDD – ADs), FDR<0.05 (MACS2), >5-fold enrichment over input –Fig. S3F-G

Supplementary Data 54: Human DRN H3K4me3Q5ser ChIP-seq peaks (Matched Controls for MDD + ADs), FDR<0.05 (MACS2), >5-fold enrichment over input –Fig. S3F-G

Supplementary Data 55: Human DRN H3K4me3Q5ser ChIP-seq peaks (MDD – ADs), FDR<0.05 (MACS2), >5-fold enrichment over input –Fig. S3F-G

Supplementary Data 56: Human DRN H3K4me3Q5ser ChIP-seq peaks (MDD + ADs), FDR<0.05 (MACS2), >5-fold enrichment over input –Fig. S3F-G

Supplementary Data 57: Human DRN H3K4me3Q5ser diffReps (MDD – ADs vs. Matched Controls All, FDR<0.05, log2FC ≥ 1.0 or ≤ –1.0 –Fig. S3F-G

Supplementary Data 58: Human DRN H3K4me3Q5ser diffReps (MDD + ADs vs. MDD – ADs, FDR<0.05, log2FC ≥ 1.0 or ≤ –1.0 –Fig. S3F-G

Supplementary Data 59: Human DRN ChIP-seq data (MDD + ADs vs. MDD – ADs) vs. Mouse DRN (Male) ChIP-seq Data (Susceptible FLX vs. Susceptible H2O) Overlap All, FDR<0.05, log2FC ≥ 1.0 or ≤–1.0, GO BP (FDR<0.05; Benjamini-Hochberg) –Fig. S3G

Supplementary Data 60: Human DRN ChIP-seq data (MDD + ADs vs. MDD – ADs) vs. Mouse DRN (Male) ChIP-seq Data (Susceptible FLX vs. Susceptible H2O) Overlap All, FDR<0.05, log2FC ≥ 1.0 or ≤–1.0, GWAS Catalog (FDR<0.05; Benjamini-Hochberg) –Fig. S3G

Supplementary Data 61: Adult Viral DRN RNA-seq data (Male Empty CSDS vs. Empty Control All; FDR<0.1; DESeq2) – Fig. 4E-F & Fig. S5A-F

Supplementary Data 62: Adult Viral DRN RNA-seq data (Male H3.3 WT CSDS vs. H3.3 WT Control All; FDR<0.1; DESeq2) – Fig. 4E

Supplementary Data 63: Adult Viral DRN RNA-seq data (Male H3.3Q5A CSDS vs. Empty CSDS Control All; FDR<0.1; DESeq2) – Fig. 4E & Fig. S6B-F

Supplementary Data 64: Adult Viral DRN RNA-seq data (Male H3.3Q5A CSDS vs. H3.3 WT CSDS Control All; FDR<0.1; DESeq2) – Fig. 4E

Supplementary Data 65: Adult Viral DRN RNA-seq data (Male Empty CSDS vs. Empty Control & H3.3Q5A CSDS vs. Empty CSDS Control Reversed; FDR<0.1), GWAS Catalog (FDR<0.05; Benjamini-Hochberg) – Fig. 4F

Supplementary Data 66: Adult Viral DRN RNA-seq data (Male Empty CSDS vs. Empty Control & H3.3Q5A CSDS vs. Empty CSDS Control Reversed; FDR<0.1), DisGeNET (FDR<0.05; Benjamini-Hochberg) – Fig. 4F

Supplementary Data 67: Adult Viral DRN RNA-seq data (Male Empty CSDS vs. Empty Control & H3.3Q5A CSDS vs. Empty CSDS Control Reversed; FDR<0.1), GO BP (FDR<0.05; Benjamini-Hochberg) – Fig. 4F

Supplementary Data 68: Adult Viral DRN RNA-seq data (Male H3.3Q5A CSDS vs. Empty CSDS) and Adult DRN (Susceptible FLX vs. Susceptible H2O) Overlap All, FDR<0.1, GO BP (FDR<0.05; Benjamini-Hochberg) –Fig. S6F

Supplementary Data 69: Adult Viral DRN RNA-seq data (Male H3.3Q5A CSDS vs. Empty CSDS) and Adult DRN (Susceptible FLX vs. Susceptible H2O) Overlap All, FDR<0.1, GWAS Catalog (FDR<0.05; Benjamini-Hochberg) –Fig. S6F

Supplementary Data 70: Human postmortem DRN demographic information - Fig. 2C & Fig. S3F-G

Supplementary Data 71: Mouse qChIP Primers - Fig. S4
