# Supplementary material for: Histone serotonylation in dorsal raphe nucleus contributes to stress- and antidepressant-mediated gene expression and behavior
Source: Nat Commun. 2024 Jun 13;15:5042. doi: 10.1038/s41467-024-49336-4 (PMC11176395; doi:10.1038/s41467-024-49336-4)
Supplement: Supplementary file 5 — Reporting Summary [file 41467_2024_49336_MOESM5_ESM.pdf]

nature portfolio

Corresponding author(s): Ian Maze

Last updated by author(s): May 9, 2024

Reporting Summary

Nature Portfolio wishes to improve the reproducibility of the work that we publish. This form provides structure for consistency and transparency in reporting. For further information on Nature Portfolio policies, see our Editorial Policies and the Editorial Policy Checklist.

Statistics

For all statistical analyses, confirm that the following items are present in the figure legend, table legend, main text, or Methods section.

n/a	Confirmed	
☐	☒	The exact sample size (*n*) for each experimental group/condition, given as a discrete number and unit of measurement
☐	☒	A statement on whether measurements were taken from distinct samples or whether the same sample was measured repeatedly
☐	☒	The statistical test(s) used AND whether they are one- or two-sided<br>*Only common tests should be described solely by name; describe more complex techniques in the Methods section.*
☒	☐	A description of all covariates tested
☐	☒	A description of any assumptions or corrections, such as tests of normality and adjustment for multiple comparisons
☐	☒	A full description of the statistical parameters including central tendency (e.g. means) or other basic estimates (e.g. regression coefficient) AND variation (e.g. standard deviation) or associated estimates of uncertainty (e.g. confidence intervals)
☐	☒	For null hypothesis testing, the test statistic (e.g. *F*, *t*, *r*) with confidence intervals, effect sizes, degrees of freedom and *P* value noted<br>*Give P values as exact values whenever suitable.*
☒	☐	For Bayesian analysis, information on the choice of priors and Markov chain Monte Carlo settings
☒	☐	For hierarchical and complex designs, identification of the appropriate level for tests and full reporting of outcomes
☒	☐	Estimates of effect sizes (e.g. Cohen's *d*, Pearson's *r*), indicating how they were calculated

Our web collection on statistics for biologists contains articles on many of the points above.

Software and code

Policy information about availability of computer code

Data collection	No custom algorithms were used.
Data analysis	RNA-SEQ AND ANALYSIS: Following RNA purification, RNA-seq libraries were prepared according to the Illumina Truseq RNA Library Prep Kit V2 (#RS-122-2001) protocol and sequenced on the Illumina Novaseq platform. Following sequencing, data was pre-processed and analyzed as previously described (19). Briefly, FastQC (Version 0.72) was performed on the concatenated replicate raw sequencing paired-end reads from each library to ensure minimal PCR duplication and sequencing quality. Reads were aligned to the mouse mm10 genome using HISAT2 (Version 2.1.0) and annotated against Ensembl v90. After removal of multiple-aligned reads, remaining reads were counted using featurecounts (Version 2.0.1) with default parameters, and filtered to remove genes with low counts (<10 reads across samples). For male 24-hr post-CSDS RNA-seq, RUVr (44), k = 6, was performed to normalize read counts based on the residuals from a first-pass GLM regression of the unnormalized counts on the covariates of interest. For female RNA-seq experiments and the serotonylation manipulation experiments with the Q5A virus, RUVr (44) (female; k = 4, Q5A; k = 6 was performed to normalized read counts. DESEQ2 (45) (Version 2.11.40.6) was used to perform pairwise differential expression analyses between indicated comparisons. Differentially expressed (DE) genes were defined at FDR<0.05. Unsupervised clustering heatmaps were generated at DE genes across samples using heatmap2 from gplots (Version 3.1.3). Threshold free Rank-Rank Hypergeometric Overlap (RRHO) maps were generated to visualize transcriptome-wide gene expression concordance patterns as previously described (46), using RRHO2 (Version 1.0). Odds ratios for overlapping gene sets were calculated with GeneOverlap (Version 1.34.0). Enrichment analysis on gene sets of interest was performed with EnrichR, Benjamini-Hochberg (BH) q-values corrected for multiple testing are reported (47–49).<br><br>CHIP-SEQ LIBRARY PREPARATION AND ANALYSIS: Following DNA purifications, ChIP-seq libraries were generated according to Illumina protocols and sequenced on an Illumina HiSeq2500, 4000 or Novaseq Sequencers. ChIP-seq peaks were called and differential analysis conducted exactly as described previously (18,50). Briefly, raw sequencing reads were aligned to the mouse or human genome (mm10 or

April 2023

hg38, respectively) using default settings of HISAT2. Alignments were filtered to only include uniquely mapped reads using SAMtools v.1.8. Peak-calling was normalized to respective inputs for each sample and was performed using MACS v.2.1.1 (51) with default settings and filtered for FDR< 0.05. Differential analysis was performed using diffReps (52) with a 1 kb window size. Peaks and differential sites were further annotated to nearby genes or intergenic regions using the region analysis tool from the diffReps package. To be considered a "real" peak-containing PCG, a significant peak (FDR<0.05, >5-fold enrichment over input) had to be found in a PCG (promoter and/or gene body) in at least one of: 3 conditions for male 24 hr post-SI testing (Control, Susceptible or Resilient); 2 conditions for female 24 hr post-SI testing (Control, Defeat); 4 conditions for fluoxetine experiments 30d post-SI testing (control -/+ FLX, SUS -/+ FLX); or 4 conditions for human DRN (MDD – ADs, MDD + ADs vs. matched controls). To be considered a differentially enriched gene, it had to first pass the aforementioned criteria, and then display a >1.5 or < –1.5 or >1.0 or < –1.0 fold difference between conditions (pairwise comparisons) at FDR<0.05 (as indicated throughout). Enrichment analysis on gene sets of interest was performed with EnrichR, Benjamini-Hochberg (BH) q-values corrected for multiple testing are reported (47–49).

For manuscripts utilizing custom algorithms or software that are central to the research but not yet described in published literature, software must be made available to editors and reviewers. We strongly encourage code deposition in a community repository (e.g. GitHub). See the Nature Portfolio guidelines for submitting code & software for further information.

Data

Policy information about availability of data

All manuscripts must include a data availability statement. This statement should provide the following information, where applicable:

- Accession codes, unique identifiers, or web links for publicly available datasets
- A description of any restrictions on data availability
- For clinical datasets or third party data, please ensure that the statement adheres to our policy

The RNA-seq and ChIP-seq data generated in this study have been deposited in the National Center for Biotechnology Information Gene Expression Omnibus (GEO) database under accession number GSE216104. We declare that the data supporting findings for this study are available within the article and Supplementary Information. No restrictions on data availability apply.

Research involving human participants, their data, or biological material

Policy information about studies with human participants or human data. See also policy information about sex, gender (identity/presentation), and sexual orientation and race, ethnicity and racism.

Reporting on sex and gender	For all de-identified postmortem studies exploring H3 serotonylation dynamics in MDD DRN, demographic information is provided in Supplemental Data Table 70
Reporting on race, ethnicity, or other socially relevant groupings	For all de-identified postmortem studies exploring H3 serotonylation dynamics in MDD DRN, demographic information is provided in Supplemental Data Table 70
Population characteristics	For all de-identified postmortem studies exploring H3 serotonylation dynamics in MDD DRN, demographic information is provided in Supplemental Data Table 70
Recruitment	Human DRN tissues from the Dallas Brain Collection (UT Neuropsychiatry Research Program) were obtained from the Southwestern Institute of Forensic Sciences at Dallas, UT Southwestern Transplant Services Center, and UT Southwestern Willed Body Program, following consent from donor subjects' next of kin, permission to access medical records and to hold direct telephone interviews with a primary caregivers. All clinical information obtained for each donor was reviewed by three research psychiatrists, using DSM-V criteria for diagnoses. Blood toxicology screens were conducted for each donor subject from the Southwestern Institute of Forensic Sciences at Dallas.
Ethics oversight	Collection of postmortem human brain tissues is approved by the University of Texas Southwestern Medical Center Institutional Review Board [STU 102010-053].

Note that full information on the approval of the study protocol must also be provided in the manuscript.

Field-specific reporting

Please select the one below that is the best fit for your research. If you are not sure, read the appropriate sections before making your selection.

☒ Life sciences ☐ Behavioural & social sciences ☐ Ecological, evolutionary & environmental sciences

For a reference copy of the document with all sections, see nature.com/documents/nr-reporting-summary-flat.pdf

Life sciences study design

All studies must disclose on these points even when the disclosure is negative.

Sample size	Adequate sample sizes are generally determined based upon inter-sample variability. Throughout the manuscript, we determined the significance of results based upon a general confidence interval of 95%. We do not include specific justifications of sample size within the

	methods (e.g., power analyses), as sample sizes were based on extensive laboratory experience with these endpoints. The sample sizes chosen are consistent with those used by others in the field to achieve statistically significant results comparing stressed vs. control animals.
Data exclusions	No data exclusions
Replication	All biological endpoints were reliably reproduced using numerous biological (>3 for all experiments in which statistics were employed) and technical replicates for each experiment. All novel tools used in this study were extensively validated, as demonstrated in the manuscript submission.
Randomization	For all behavioral and genomic analyses, animals were randomly assigned to groups (segregated by viral treatments and/or stress exposures). Tissue samples for biochemical and RNA-seq assessments were not pooled from multiple animals in these studies (i.e., each n represents a discrete data point). Pooling per group was performed for ChIP-seq experiments, as indicated throughout the manuscript, to obtain enough input material for optimal sequencing results.
Blinding	For all behavioral, biochemical and genomic analyses, investigators were blinded to conditions such viral treatment etc. prior to analysis.

Reporting for specific materials, systems and methods

We require information from authors about some types of materials, experimental systems and methods used in many studies. Here, indicate whether each material, system or method listed is relevant to your study. If you are not sure if a list item applies to your research, read the appropriate section before selecting a response.

Materials & experimental systems

n/a	Involved in the study	
☐	☒	Antibodies
☒	☐	Eukaryotic cell lines
☒	☐	Palaeontology and archaeology
☐	☒	Animals and other organisms
☒	☐	Clinical data
☒	☐	Dual use research of concern
☒	☐	Plants

Methods

n/a	Involved in the study	
☐	☒	ChIP-seq
☒	☐	Flow cytometry
☒	☐	MRI-based neuroimaging

Antibodies

Antibodies used	WESTERN BLOTTING AND CHIP-SEQ - The following antibodies were used: rabbit anti-H3K4me3Q5ser (1:500, ABE2580; MilliporeSigma), rabbit anti-H3Q5ser (1:500, MilliporeSigma; ABE1791,), rabbit anti-H3 (1:50000, Abcam ab1791), H4 (1:10000, Abcam; ab10158), H3.3 (1:2000, MilliporeSigma; 09-838,), FLAG (1:5000, Sigma; F3165,) and rabbit anti-Gapdh (1:10000, Abcam; ab9485).<br><br>IMMUNOHISTOCHEMISTRY - rabbit anti-H3K4me3Q5Ser (1:500, MilliporeSigma; ABE1791), mouse anti-HA (1:1000 , SantaCruz, Cat#, sc-7392; donkey anti-rabbit AlexaFluor568 – ThermoFisher A-10042 - and donkey anti-mouse AlexaFluor680 – ThermoFisher A-21109; 1:1000 & DAPI (1:10000, Thermo Scientific 62248)
Validation	Antibodies used in this study (all of which have been commercially validated by the manufacturers) were validated in human/rodent cells/tissues via immunoblotting, IPs or ICC/IHC/IF prior to experimentation (analyzed for correct migration size, sub-cellular localization patterns, etc.).

Animals and other research organisms

Policy information about studies involving animals; ARRIVE guidelines recommended for reporting animal research, and Sex and Gender in Research

Laboratory animals	C57BL/6J mice were purchased from The Jackson Laboratory. Retired male CD-1 breeders of at least 4 months of age were purchased from Charles River laboratories and used as aggressors. Female Swiss Webster (CFW) mice were purchased from Charles River laboratories. All mice were singly housed following CSDS and maintained on a 12-h/12-h light/dark cycle throughout the entirety of the experiments. Mice were provided with ad libitum access to water and food throughout the entirety of the experiments. Mouse housing room conditions: 72F, 50% humidity.
Wild animals	The study did not involve samples collected from wild animals.
Reporting on sex	Both male and female mice were assessed in these studies.
Field-collected samples	The study did not involve samples collected from the field.
Ethics oversight	All animal procedures were done in accordance with NIH guidelines and with approval with the Institutional Animal Care and Use Committee of the Icahn School of Medicine at Mount Sinai.

Note that full information on the approval of the study protocol must also be provided in the manuscript.

Plants

Seed stocks	N/A
Novel plant genotypes	N/A
Authentication	N/A

ChIP-seq

Data deposition

☒ Confirm that both raw and final processed data have been deposited in a public database such as GEO.

☒ Confirm that you have deposited or provided access to graph files (e.g. BED files) for the called peaks.

Data access links<br>*May remain private before publication.*	The RNA-seq and ChIP-seq data generated in this study have been deposited in the National Center for Biotechnology Information Gene Expression Omnibus (GEO) database under accession number GSE216104 (Reviewer Token: cfsnggochnatbqv).
Files in database submission	S1, S2, S3, S4, S5, S6, S7, S8, S9, S10, S11, S12, S13, S14, S15, S16, S17, S18, S19, S20, S21, S22, S23, S24, C1_2844_S2, C3_2841_S6, C4_2845_S8, C5_2842_S10, C6_2843_S12, D1_2840_S1, D2_2849_S3, D3_2851_S5, D4_2852_S7, D5_2847_S9, D6_2850_S11, CG1, CG2, CG3, CG4, CQ1, CQ2, CQ3, CW1, CW2, SG1, SG11, SG3, SG4, SQ1b, SQ11, SQ2, SQ3, SW1, SW2, SW3, SW4, CG5, CG6, CG7, CQ4, CQ5, CQ6, CQ7, SG5, SG6, SG9, SG7, SQ7, SQ8, SW11, SW5, SW6, SW9, 12_S8_L005_R1_001, 12_S8_L006_R1_001, 12_S8_L007_R1_001, 15_S9_L005_R1_001, 15_S9_L006_R1_001, 15_S9_L007_R1_001, 16_S6_L005_R1_001, 16_S6_L006_R1_001, 16_S6_L007_R1_001, 19_S7_L005_R1_001, 19_S7_L006_R1_001, 19_S7_L007_R1_001, 2_S1_L005_R1_001, 2_S1_L006_R1_001, 2_S1_L007_R1_001, 4_S2_L005_R1_001, 4_S2_L006_R1_001, 4_S2_L007_R1_001, 5_S3_L005_R1_001, 5_S3_L006_R1_001, 5_S3_L007_R1_001, 6_S4_L005_R1_001, 6_S4_L006_R1_001, 6_S4_L007_R1_001, 7_S5_L005_R1_001, 7_S5_L006_R1_001, 7_S5_L007_R1_001, 1-input_S17_L005_R1_001, 1_S23_L006_R1_001, 2-input_S18_L005_R1_001, 2_S24_L006_R1_001, 3-input_S19_L005_R1_001, 3_S25_L006_R1_001, 4-input_S20_L005_R1_001, 4_S26_L006_R1_001, 5-input_S21_L005_R1_001, 5_S27_L006_R1_001, 6-input_S22_L005_R1_001, 6_S28_L006_R1_001, C41_S14_L002, C43_S5_L002, C44_S15_L002, C45_S13_L002, C46_S18_L002, C48_S20_L002, C49_S17_L002, C59_S19_L002, C61_S6_L002, C64_S16_L002, D43_S3_L002, D46_S9_L002, D53_S8_L002, D63_S1_L002, D64_S7_L002, D65_S11_L002, D86_S10_L002, D88_S12_L002, D91_S2_L002, 201input_S27_L005, 202input_S46_L007, 203input_S49_L007, 204input_S32_L005, 205input_S26_L005, 206input_S31_L005, 207input_S51_L007, 208input_S29_L005, 209input_S52_L007, 210input_S34_L005, 211input_S54_L007, 212input_S33_L005, 213input_S55_L007, 214input_S50_L007, 215input_S30_L005, 216input_S47_L007, 217input_S35_L005, 218input_S48_L007, 219input_S28_L005, 220input_S53_L007, 201_S48_L006, 202_S36_L006, 203_S39_L006, 204_S53_L006, 205_S47_L006, 206_S52_L006, 207_S41_L006, 208_S50_L006, 209_S42_L006, 210_S55_L006, 211_S44_L006, 212_S54_L006, 213_S45_L006, 214_S40_L006, 215_S51_L006, 216_S37_L006, 217_S56_L006, 218_S38_L006, 219_S49_L006, 220_S43_L006, CF1_input, CF1_IP, CF2_input, CF2_IP, CF3_input, CF3_IP, CH1_input, CH1_IP, CH2_input, CH2_IP, CH3_input, CH3_IP, SF1_input, SF1_IP, SF2_input, SF2_IP, SF3_input, SF3_IP, SH1_input, SH1_IP, SH2_input, SH2_IP, SH3_input, SH3_IP
Genome browser session<br>(e.g. UCSC)	https://ramaka02.dmz.hpc.mssm.edu/UCSC-TrackHub-Human-Ian-Sept2023/<br>https://ramaka02.dmz.hpc.mssm.edu/UCSC-TrackHub-Mouse-Ian-Sept2023/

Methodology

Replicates	Biological replicates were used in all genomics analyses and are reported in the Figures Legends of the manuscript.
Sequencing depth	Average sequencing depth for RNA-seq experiments: 36 Million reads<br>Average sequencing depth for ChIP-seq experiments: 32 Million reads
Antibodies	Samples were incubated with a custom rabbit anti-H3K4me3Q5ser (1:500, ABE2580; MilliporeSigma).
Peak calling parameters	Peak-calling was normalized to respective inputs for each sample and was performed using MACS v.2.1.1 (51).
Data quality	Peaks were filtered for FDR < 0.05 and fold change > 5.0. All processed peak data, along with respective fold changes and FDR values, are provided in the Extended Data Tables as part of this initial submission.
Software	HISAT2<br>MACSv2.1.1<br>diffReps<br><br>Threshold free Rank-Rank Hypergeometric Overlap (RRHO) maps were generated to visualize transcriptome-wide gene expression concordance patterns as previously described (46), using RRHO2 (Version 1.0). Odds ratios for overlapping gene sets were calculated with GeneOverlap (Version 1.34.0). Enrichment analysis on gene sets of interest was performed with EnrichR, Benjamini-Hochberg (BH) q-values corrected for multiple testing are reported (47–49).
